# Assessment of P2Y_12_ Inhibition by Clopidogrel in Feline Platelets Using Flow Cytometry Quantification of Vasodilator-Stimulated Phosphoprotein Phosphorylation

**DOI:** 10.3389/fvets.2020.00267

**Published:** 2020-05-27

**Authors:** Ronald H. L. Li, Nghi Nguyen, Tommaso Rosati, Karl Jandrey

**Affiliations:** ^1^Department of Veterinary Surgical and Radiological Sciences, School of Veterinary Medicine, University of California, Davis, Davis, CA, United States; ^2^William R. Pritchard Veterinary Medical Teaching Hospital, University of California, Davis, Davis, CA, United States

**Keywords:** hypertrophic cardiomyopathy, platelet activation and signaling, light transmission aggregometry, vasodilator stimulated phosphoprotein, cyclic AMP

## Abstract

The primary objective of this study was to evaluate a novel flow cytometry-based assay of quantifying platelet phosphorylation of vasodilator-stimulated phosphoprotein (P-VASP) in cats that received clopidogrel treatment. Eight healthy cats received 18.75 mg PO q24h of clopidogrel for 7 days. Prior to and after clopidogrel treatment, blood was collected for ADP-induced light transmission aggregometry (LTA) and P-VASP measurement by flow cytometry. Flow cytometry measurement of P-VASP levels was used to derive platelet reactivity index (PRI) before and after clopidogrel treatment. Based on P-VASP and LTA findings, platelet response to ADP was significantly attenuated after 7 days of clopidogrel treatment. By eliciting the competing platelet pathways of P2Y_12_ and cAMP using ADP and PGE_1_, respectively, ADP had no effect on P-VASP levels following clopidogrel treatment (*p* = 0.94). Clopidogrel also significantly decreased PRI from 28.84 ± 28.52% to 1.69 ± 12.39% (*p* = 0.0078). PRI on day 8 correlated moderately with the degree of slope inhibition on LTA (*r* = −0.4, *p* = 0.4). Flow cytometry analysis of P-VASP is effective at monitoring the inhibitory effects of clopidogrel on feline platelets.

## Introduction

Hypertrophic cardiomyopathy (HCM) is the most common cardiomyopathy in cats affecting ~15% of the feline population (1). Since cardiogenic arterial thromboembolism (CATE) is one of the most devastating sequelae of HCM in cats, anti-platelet drugs like aspirin and clopidogrel are the cornerstone of anticoagulant therapy in these cats (2–5). Clopidogrel, once metabolized to its active metabolite by hepatic cytochrome P450, inhibits ADP-mediated platelet activation by covalently binding onto one of the two G protein-coupled ADP receptors, P2Y_12_. This irreversibly prevents ADP from activating the G protein, G_i2a_, responsible for downstream signaling that amplifies platelet activation and aggregation (6). A randomized controlled trial in cats with CATE demonstrated the superiority of clopidogrel over aspirin in reducing recurrent thrombotic events (7). Despite this favorable outcome, recurrence of CATE remains high, thus, underscoring the highly variable pharmacodynamic response to clopidogrel in clinical HCM cats. Because clopidogrel resistance has long been associated with on-treatment thrombotic events in human patients, a reliable and accurate assay to measure clopidogrel response in cats is needed.

In response to P2Y_12_ inhibition or prostaglandin E_1_ (PGE_1_), platelet cyclic adenosine monophosphate (cAMP) is elevated resulting in protein kinase A activation and subsequent phosphorylation of the actin regulatory protein, vasodilator-stimulated phosphoprotein (VASP) (8). Conversely, platelet activation by the agonist, ADP, inhibits cAMP production and dephosphorylation of VASP. The degree of VASP phosphorylation (P-VASP) within platelets, therefore, serves as a marker of P2Y_12_ activation or inhibition (9, 10). The authors had previously evaluated P-VASP in feline platelets using Western blot analysis. Although a significant increase in P-VASP was found in response to clopidogrel therapy, interindividual variability was extremely high (11). In addition, protein analysis by Western blot only yielded semi-quantitative results that were subjected to high inter-operator variability. For that reason, intraplatelet P-VASP analysis by quantitative flow cytometry has been extensively studied in human beings. In human studies, P-VASP quantification by flow cytometry has been shown to be sensitive in monitoring the inhibitory effects of P2Y_12_ antagonists (9, 12). However, quantification of platelet P-VASP by flow cytometry as a marker of inhibitory and stimulatory pathways has never been evaluated in cats.

Herein, we described a new flow cytometry-based method to measure the inhibitory effects of clopidogrel in cats by quantifying P-VASP within feline platelets. We also aimed to evaluate the pharmacodynamic response to clopidogrel by correlating flow cytometric measurement of platelet P-VASP to ADP-induced LTA in cats treated with 7 days of clopidogrel. We hypothesized that clopidogrel treatment in cats would lead to intracellular inhibitory pathways resulting in phosphorylation of VASP and that the degree of platelet VASP phosphorylation would correlate with inhibition in ADP-induced aggregation as measured by LTA.

## Materials and Methods

### Animals and Study Design

The study protocol was approved by the Institutional Animal Care and Use Committee at the University of California, Davis (IACUC #20359). Eight university-owned, domestic shorthaired cats from the Feline Nutrition and Pet Care Center at University of California, Davis were used in this study. The mean age of cats was 4.5 years (range: 2–5 years). All cats were female spayed Domestic Shorthair cats. All cats were not enrolled in other studies at the time of data collection and were deemed to be clinically healthy based on physical examinations performed by the authors prior to the study. Prior to clopidogrel therapy (day 0), 4 ml of whole blood was collected from the jugular vein or medial saphenous vein using a 20-gauge needle or 21-gauge butterfly catheter, and, immediately aliquoted to 3.2% trisodium citrate tubes. Each cat then received 18.75 mg clopidogrel bisulfate (1/4 of a 75 mg Plavix® tablet, Britol-Myers Squibb/Sanofi Pharmaceuticals, Bridgewater, NJ) every 24 h PO for 7 days. The tablet was administered to each cat using a pilling device to insert the tablet into the oral cavity over the base of the tongue. Cats were then observed to swallow to confirm that the dose was administered. All cats were monitored closely for adverse effects such as vomiting, lethargy, inappetence, diarrhea and bleeding diathesis throughout the study period. Blood was drawn, as described above, ~12 h after the last dose of clopidogrel (Day 8).

### Generation of Platelet Rich Plasma

Following thorough mixing of anticoagulant, blood was transferred to round-bottom polypropylene tubes and placed in 37°C bead bath for 30 min to facilitate sedimentation of erythrocytes. Platelet rich plasma (PRP) was then generated by centrifugation at 200 x g for 5 min (no brakes, 25–27°C). Complete blood count of whole blood and platelet count of PRP were obtained using an automated analyzer (HM5, Abaxis, Union City, CA) and confirmed by blood smear evaluation. All samples were analyzed within 2 h after collection.

### Cell Preparation and Immunolabelling

Platelet concentration in PRP was standardized to 2 × 10^7^/ml with Tyrodes HEPES (pH 7.2, 5 mM dextrose without divalent cations) to a final volume of 100 μl (11). Platelets were treated with 10 μM PGE_1_ (MilliporeSigma, Burlington, MA), as positive control, 20 μM ADP (MilliporeSigma, Burlington, MA), or with a combination of ADP and PGE_1_ for 15 min at 37°C. Platelets were then fixed in 1% methanol-free paraformaldehyde for 15 min at room temperature. Immediately after permeabilization using 0.25% detergent (NP-40 Surface-AMPs Detergent Solution, ThermoFisher), PRP was subjected to centrifugation at 5,000 × g for 1 min at room temperature. Supernatant was immediately discarded, and pellets were resuspended with 100 μl Tyrodes HEPES (no divalent cations). Phosphorylation at serine-239 of VASP was detected using a mouse polyclonal antibody conjugated to fluorescein isothiocyanate (5 ug/mL, ALX-804-240F-C100, Enzo Life Sciences, Farmingdale, NY) for 90 min at room temperature (protect from light). The amino acid sequence of P-VASP is highly homologous when compared to the published feline VASP protein sequence as determined by the Basic Local Alignment Search Tool, BLAST (National Institute of Health).

### Flow Cytometry

**Figure 1:**
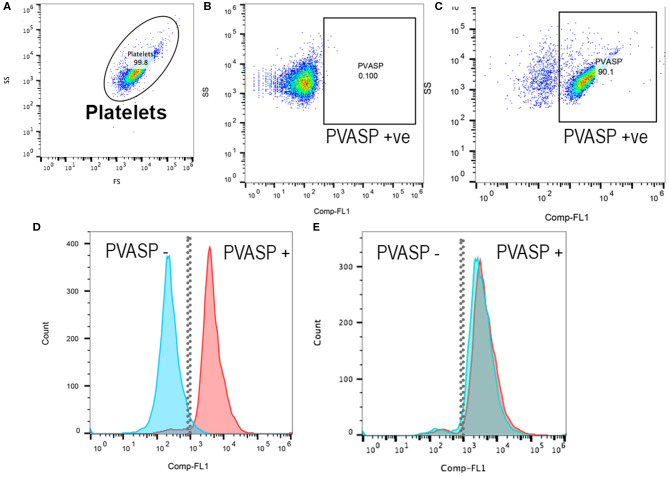
Representative scatter plot diagrams and histograms of flow cytometric analysis of intraplatelet vasodilator-stimulated phosphoprotein phosphorylation in a cat before and after clopidogrel therapy. **(A)** Platelets were identified based on their forward (FS) and side scatter (SS) properties. **(B)** Fluorescent minus one control and isotype control in PGE1-treated platelets were used to determine the P-VASP gate. P-VASP positive platelets were labeled as P-VASP +ve. **(C)** In PGE_1_-treated platelets, 90.1% of platelets expressed the phosphorylated form of vasodilator-stimulated phosphoprotein (P-VASP). **(D)** A histogram illustrating the number of platelets expressing P-VASP in PGE1-treated platelets in presence (Blue) or absence of ADP (Red) prior to clopidogrel therapy. Note that P2Y_12_ activation by ADP resulted in loss of P-VASP as the population shifts to the left. **(E)** Following clopidogrel therapy, regardless of ADP activation, PGE_1_ resulted in VASP phosphorylation due to irreversible inhibition of P2Y_12_.

Platelets were analyzed within 2 h following fixation, permeabilization and labeling. PRP were further diluted (1:10) with Tyrodes HEPES before filtering through a cell strainer prior to analysis. Flow cytometry was performed using a 5-color flow cytometer (Beckman-Coulter FC500 Flow Cytometer, Beckman-Coulter Inc). Platelets were identified by forward- and side-scatter properties as previously described (11) using 0.9 and 3 μm calibration beads and each sample was analyzed until 10,000 events were recorded (Figure 1A). In brief, gating of P-VASP-positive events was established by isotype and fluorescence-minus-one controls consisting of unlabeled cells that were subjected to the same experimental conditions (Figures 1B,C). Mean fluorescence intensity (MFI) of P-VASP positive platelets (Figures 1D,E) were measured using commercially available software (Flowjo, Ashland, Oregon).

### Light Transmission Aggregometry

PRP generated from citrated whole blood was diluted to 1.5 × 10^8^/ml using Tyrodes-HEPES (pH 7.2, 5 mM dextrose, no divalent cations). Platelet poor plasma, generated by centrifugation at 10,000 × g for 5 min, from each cat served as the control light transmission. PRP was aliquoted to warmed siliconized cuvettes (Chronolog, Havertown, PA) containing a magnetic stir bar set at a constant stir speed of 1,200 rpm. Aggregation was then recorded for 1 min as baseline before the addition of 40 μM ADP or 1 U/ml bovine α-thrombin (Haematologic Technologies, Essex Junction, VT), utilized as positive control. Aggregation was recorded for an additional 5 min and measured as percent (%) of maximum aggregation, slope and area under the curve (AUC) transmission using commercially available software (Chronolog, Havertown, PA), as previously validated for use in cats (11).

### Response to Clopidogrel

The inhibitory effect of clopidogrel on platelets was categorized based on previously established criteria (11). Percent (%) inhibition by clopidogrel based on ADP-induced aggregation (ADP-Ag), was measured by % aggregation, slope and AUC, was calculated using Equation (1).

(1) $$\begin{array}{l} \text{Percent inhibition}(\%)\;=\\ \frac{\left(\text{ADP​}-\text{​Ag}{\;}_{\text{Day0}}\;-\text{ ADP​}-\text{​Ag}{\;}_{\text{Day8}}\right)}{\text{ADP}-\text{Ag}{\;}_{\text{Day0}}}\;\times \text{100} \end{array}$$

For the purpose of this study, cats with <60% inhibition were classified as non-responders (11). The magnitude of P2Y_12_ inhibition was measured by the platelet reactivity index (PRI), calculated based on P-VASP MFI using Equation (2) (13):

(2) $$\text{PRI}=\left[\frac{\left({\text{MFI}}_{\text{PGE1}}-{\text{MFI}}_{\text{PGE1+ADP}}\right)}{{\text{MFI}}_{\text{PGE1}}}\right]\times \text{100}$$

### Statistical Analysis

Normality of data was tested using Shapiro-Wilk normality test or visual inspection of normal quartile plots. Non-parametric data were presented as median and interquartile range (IQR) and parametric data were presented as mean ± standard deviation. Normally distributed and paired data were analyzed using paired *t*-tests while nonparametric and paired data were analyzed using Wilcoxon signed-rank test. To evaluate correlation between P-VASP PRI and LTA findings, Spearman correlation and correlation coefficient were calculated using standardized units [Standardized × = (×-mean)/standard deviation]. An alpha-priori of <0.05 was considered statistically significant. Interindividual variability was calculated as the ratio between the standard deviation of a group and its means and expressed as coefficient of variation (CV). Data were analyzed using commercially available software (Prism 8.0, GraphPad Software, La Jolla, CA).

## Results

No adverse effects secondary to clopidogrel were observed throughout the study period. Mean platelet count on day 0 was 310 × 10^9^/ml ± 62, which was significantly lower than platelet count on day 8 (415 × 10^9^/ml ± 103, *p* = 0.0032) following clopidogrel therapy.

### Flow Cytometric P-VASP Analysis

**Figure 2:**
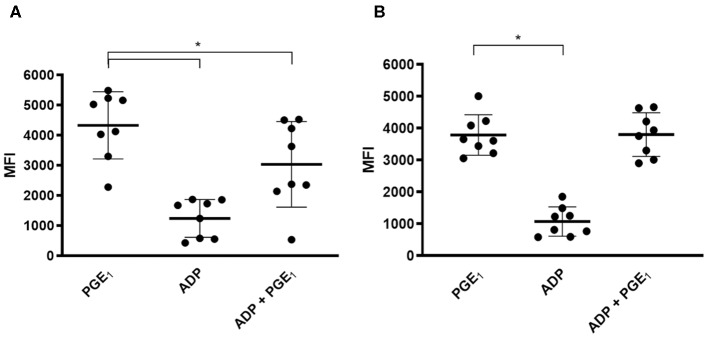
Scatter dot plots of intraplatelet vasodilator-stimulated phosphoprotein phosphorylation (P-VASP) in eight cats before and after 7 days of clopidogrel therapy. Platelet rich plasma from eight cats treated with PGE1, ADP or a combination of PGE1 and ADP was evaluated for P-VASP expression, as quantified by mean fluorescence intensity (MFI) using flow cytometry. **(A)** Before clopidogrel therapy, ADP-induced platelet activation in the presence of PGE_1_ resulted in significant decrease in P-VASP expression compared to PGE-1 treated platelets in the absence of ADP. **(B)** Following clopidogrel therapy, ADP in the presence of PGE_1_ did not result in significant decrease in P-VASP expression compared to PGE_1_-treated platelets. ADP-induced platelet activation resulted in significant decrease in P-VASP expression regardless of clopidogrel treatment. Middle line represents the median and upper and lower lines represent the 25th and 75th quartile, respectively. *p < 0.05.

On day 0, compared to PGE_1_-treated platelets, ADP-activated platelets expressed significantly lower levels of P-VASP (4,332 ± 1,115 vs. 1,245 ± 628.9, *p* < 0.0001). PGE_1_ in the presence of ADP also resulted in significantly lower P-VASP levels (3,038 ± 1,420 vs. 4,332 ± 1,115, *p* = 0.026) (Figure 2A). After 7 days of clopidogrel treatment, P-VASP in PGE_1_-treated platelets was not significantly different from P-VASP in platelets treated with ADP and PGE1-treated platelets (MFI = 3,787 ± 633.5 vs. 3,803 ± 686.6, *p* = 0.94). ADP-treated platelets continued to have significantly lower P-VASP compared to PGE_1_-treated platelets (1,072 ± 458.8 vs. 3,787 ± 633.5, *p* < 0.0001) (Figure 2B).

### Light Transmission Aggregometry

**Figure 3:**
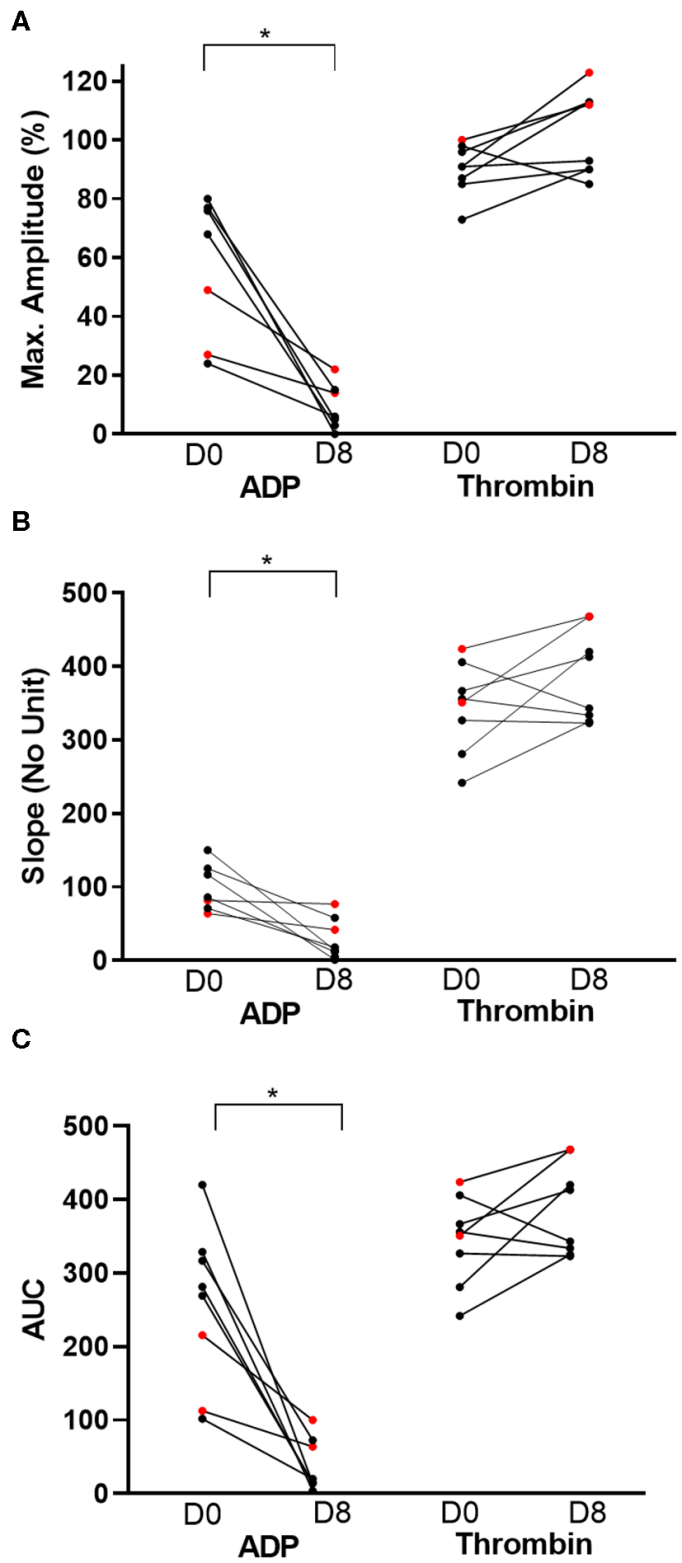
Before-and-after dot plots of light transmission aggregometry from eight cats before and after 7 days of clopidogrel treatment. ADP or thrombin-induced platelet aggregometry was measured as maximum amplitude (%), slope (no unit) and area under the curve (AUC). Thrombin-induced platelet aggregation served as positive control. ADP-induced aggregation, as measured by percent of maximum amplitude **(A)**, slope **(B)** and AUC **(C)**, was significantly decreased on day 8 (D8) compared to day 0 (D0). In contrast, thrombin did not result in significant changes in all three measurements on light transmission aggregometry. Red dots represent cats that were resistant to clopidogrel therapy. *p < 0.05.

On day 0, the mean ADP-Ag was 57.29 ± 24.03% and was significantly decreased following 7 days of clopidogrel treatment (8.75 ± 7.44%, *p* = 0.0038) (Figure 3A). Thrombin, as positive control, did not induce significant differences in aggregation between days 0 and 8 (90.13 ± 8.66% vs. 102.4 ± 14.34%, *p* = 0.05). Similarly, slopes and AUC were significantly different decreased after 7 days of clopidogrel treatment (AUC: 255.90 ± 108.6 vs. 37.91 ± 35.74, *p* = 0.0017, Slope: 99.29 ±31.80 vs. 28.38 ± 27.53, *p* = 0.0090) (Figures 3B,C).

### Variability in Clopidogrel Response

**Figure 4:**
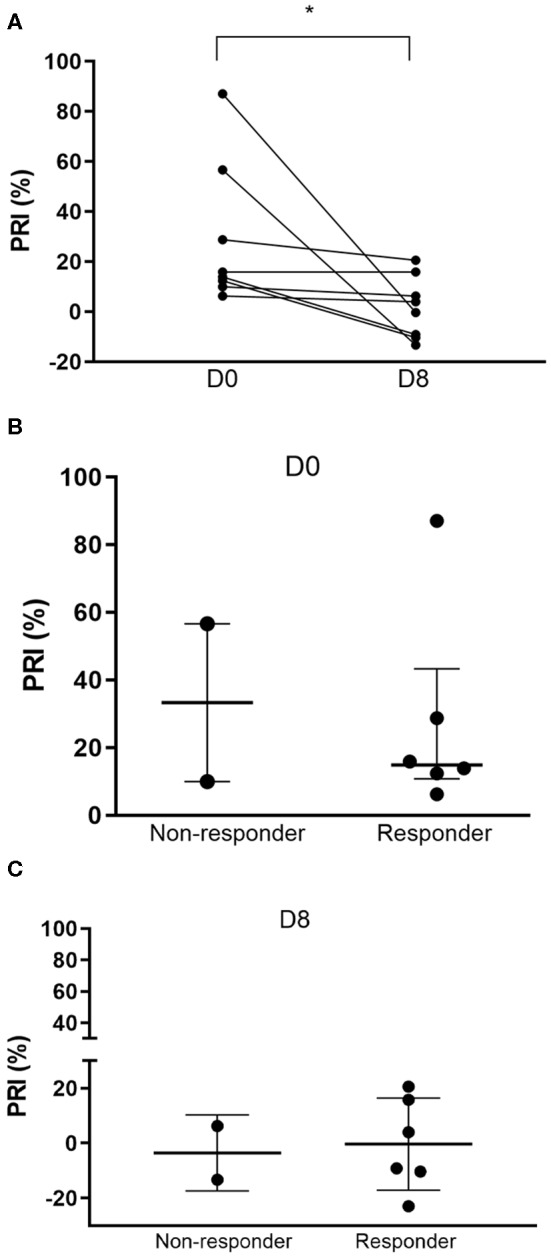
Platelet reactivity index (PRI) based on flow cytometric quantification of intraplatelet P-VASP. **(A)** Before-and-after dot plots of PRI in 8 cats following 7 days of clopidogrel treatment. PRI was significantly lower after clopidogrel treatment. **(B)** Prior to clopidogrel treatment (Day 0), PRI appeared to be higher among non-responders compared to responders. **(C)** However, on day 8, PRI was similar among the two groups. *p < 0.05.

The mean PRI based on P-VASP levels on day 0 was 28.84 ± 28.52%. The mean PRI after 7 days of clopidogrel therapy was significantly lower at 1.69 ± 12.39% (*p* = 0.0078) (Figure 4A). Standard deviations of PRI measured by P-VASP on days 0 and 8 were 28.52 and 12.39%, respectively. Coefficient of variations were 98.91% (range: 6.22–87.04%) and 732.93% (range: −13.33 to 20.57%) on days 0 and 8, respectively.

Based on LTA, 3/8 cats (37.5%) were identified as non-responders with percent inhibition of 48.1, 55.0, and 6% (Figure 3). The median percent inhibition among responders was 93.42% (IQR: 77.76–97.79). Prior to clopidogrel therapy, the median PRI among the three non-responders was 56.66% (IQR: 9.97–87.04). Responders, on the other hand, had a median baseline PRI of 13.84% (IQR: 9.29–22.30) (Figure 4B). Median PRI was −13.33% (IQR: −22.95 to 6.252) and 3.983% (IQR: −9.744 to 18.21) among the three non-responders and responders, respectively, following clopidogrel therapy (Figure 4C).

### Correlation Between ADP-Induced Light-Transmission Aggregometry and Platelet Reactivity Index Based on Flow Cytometric P-VASP

The correlations between ADP-induced LTA and PRIs on days 0 and 8 are summarized in Table 1. PRI based on P-VASP correlated moderately, but not significantly, with percent inhibition of slope based on ADP-induced LTA on day 8.

**Table 1:** Correlation between platelet reactivity index based on flow cytometric P-VASP and ADP-induced light transmission aggregometry.

	**PRI (P-VASP)**			
	**Day 0**		**Day 8**	
	***r*-value**	***p*-value**	***r*-value**	***p*-value**
**LTA (% inhibition)**				
Amplitude	−0.46	0.30	−0.089	0.90
Slope	−0.11	0.83	−0.40	0.40
AUC	−0.19	0.66	−0.089	0.90

## Discussion

This is the first study to report the use of flow cytometry quantification of platelet P-VASP in healthy cats following clopidogrel treatment. We demonstrated that P2Y_12_ activation by ADP significantly diminishes platelet P-VASP levels in the presence of PGE_1_. On the other hand, irreversible inhibition of P2Y_12_ by clopidogrel results in sustained phosphorylation of VASP when platelets are in the presence of PGE_1_ and ADP. We also found that PRI based on platelet P-VASP following 7 days of clopidogrel treatment in cats had a moderate but insignificant correlation with ADP-induced LTA.

ADP-mediated platelet activation via P2Y_12_ and platelet inhibition by endothelial factors like prostacyclin, prostaglandin or nitric oxide are competing pathways regulated by cAMP (8, 10). P2Y_12_ antagonists like clopidogrel active metabolite offsets this balance by modulating the activation of adenyl cyclase and subsequently increasing cAMP production, which inhibits platelet function (14, 15). We elicited these competing cAMP-regulated pathways by treating platelets with ADP and PGE_1_ simultaneously. Measurement of phosphorylation of VASP, a substrate of cAMP-dependent kinases like protein kinase A, therefore, can be a marker of platelet inhibition and, specifically, P2Y_12_ antagonism. In agreement with our previous study, which utilized protein electrophoresis and Western blot analysis in platelet lysate, we were able to detect irreversible P2Y_12_ inhibition in clopidogrel-treated cats by measuring intraplatelet P-VASP levels within platelets using flow cytometry (11).

Flow cytometry has been used in cats to evaluate surface protein expression, platelet-leukocyte interaction, integrin activation and the shedding of platelet-derived microvesicles (11, 16). However, it has never been used to evaluate protein phosphorylation within feline platelets. In contrast to gel-electrophoresis and Western blot analysis, flow cytometry does not require multiple blocking and immunolabeling steps, both of which can be labor intensive and time consuming. Still, intracellular protein detection by flow cytometry requires fixation and cell membrane permeabilization prior to the immunolabelling step. Fixation of platelets by paraformaldehyde or formaldehyde leads to crosslinking of methylene bridges which stabilizes and immobilizes intracellular proteins. We recommend using methanol-free paraformaldehyde as the oxidation of formaldehyde can generate formic acid and ketones that may lead to artifact and autofluorescence during analysis (17). Prolonged fixation or fixation using high concentrations of paraformaldehyde can also mask the antigen of interest preventing the binding of fluorochrome-conjugated antibodies (18). For antibodies to detect intracellular proteins, the cell membrane must first be permeabilized with a detergent. We found that brief permeabilization in low concentration of detergent (<0.5%), followed by a quick washing step, prevents significant alteration of the light scatter profiles of platelets and autofluorescence as a result of micelle formation and loss of the cytoplasm (Figure 1). Alternatively, permeabilization can be achieved using ice cold methanol.

LTA is considered the “gold standard” for monitoring anti-platelet therapy, although this has not been consensually recommended in veterinary medicine (19). To determine if flow cytometry quantification of P-VASP corresponds with LTA in cats, we calculated the correlation between PRI based on P-VASP and changes in LTA parameters before and after clopidogrel treatment. Despite the small number of cats in this study, we found moderate but insignificant correlation between P-VASP PRI and slope inhibition based on LTA. The slope of LTA tracings is calculated based on the rate of platelet aggregation in response to a platelet agonist. In the presence of low concentrations of ADP or P2Y_12_ inhibition, the rate of aggregation or slope fluctuates as platelets oscillate between aggregation and de-aggregation states due to the insufficient amount of ADP to trigger full aggregation (20). Without concurrent activation of P2Y_12_, activation of P2Y_1_ by ADP results in elevation in cytosolic calcium but insufficient shape change and activation of the integrin, α_IIb_β_3_, to sustain aggregation (21). This may result in decreases in slope of LTA and PRI following clopidogrel treatment.

Like previous studies in cats, we found a high interindividual variability in response to a standard dose of clopidogrel. The proportion of clopidogrel non-responders in this study is also similar to a different population of cats with the genetic predisposition to HCM (11, 22, 23). Based on a study by Lee et al. (23), this pharmacodynamic variability could be explained by nonsynonymous single nucleotide polymorphisms in the feline *CYP2C* genes, responsible for encoding the cytochrome P isoenzymes for metabolizing clopidogrel to its active metabolite. However, no studies to date have documented the phenotypes of these mutations in cats based on clopidogrel-specific platelet function assays. We found that low responders may have an exaggerated response to ADP as demonstrated by elevated PRI prior to clopidogrel therapy, suggesting that mutations of other genes like *P2RY12* and *P2RY1*, which encode the platelet ADP purinergic receptors, may play a role in clopidogrel resistance in cats. Because changes in P-VASP are specific at monitoring the inhibitory effects of clopidogrel, further studies using flow cytometry analysis of P-VASP are needed to assess the impact of genetic polymorphisms on the pharmacodynamics of clopidogrel in cats.

Given the small number of cats in this study, we were unable to identify a cut-off of PRI to differentiate between responders and non-responders after clopidogrel treatment. On the other hand, we found a trend suggesting that responders and non-responders had similar PRI following clopidogrel treatment. There are several explanations to this observation. First, for the purpose of this study, clopidogrel response is empirically categorized based on previously established guidelines. It is important to note that LTA and P-VASP have different specificities as LTA also tests for other ADP activation pathways. For that reason, there is also poor agreement among P-VASP and LTA in identifying poor responders to clopidogrel in human beings (24). Second, activation of cAMP and subsequent phosphorylation of VASP in platelets are proportional to the concentration and duration of exposure to PGE_1_ (9). The dose and duration of PGE_1_ used in this study may have overwhelmed the competing pathway elicited by P2Y_12_ activation in the post-clopidogrel period. Further titration for the optimal concentration and duration of PGE_1_ treatment is needed to improve the sensitivity of this assay.

Lastly, this study has a few limitations. First, we did not evaluate the within-laboratory precision or repeatability of this assay due to the limited volume of blood samples collected for the study. Further studies examining the precision and repeatability of cytometry quantification of P-VASP in feline platelets are needed. In addition, the high concentration of PGE_1_, previously utilized to evaluate P-VASP by Western blot analysis, is a limitation. Lastly, only female cats were included in this study. It has been well established that there are gender differences in platelet function in human beings; men have greater platelet adhesion and spreading while women have higher platelet reactivity in the presence of exogenous agonists (25). The evidence of gender differences in response to antiplatelet therapy, however, is inconclusive and likely complicated by fluctuations of androgens and other sex hormones. The fact that male cats are nearly twice as likely to be affected by CATE as female cats suggests that gender differences in platelet function may occur in cats (3, 26). Another study also found that female cats had higher clopidogrel active metabolite than male cats after a single dose of clopidogrel highlighting the gender differences in cytochrome P450 activity (23). Future studies with a population of cats that reflects the signalment of cats at risk of CATE are needed.

## Conclusion

Our study shows that analysis of intraplatelet P-VASP by flow cytometry is feasible in feline platelets. This assay can be a useful tool in analyzing the therapeutic efficacy of P2Y_12_ antagonists like clopidogrel as well as dysfunction in receptors and platelet signaling.

## Data Availability Statement

The raw data supporting the conclusions of this article will be made available by the authors, without undue reservation, to any qualified researcher.

## Ethics Statement

The animal study was reviewed and approved by Institutional Animal Care and Use Committee, University of California, Davis.

## Author Contributions

RL, KJ, and TR secured funding for this study. RL, NN, KJ, and TR participated in designing the study, sampling of animals, and collection of data. RL and NN performed analysis of the results. RL drafted the manuscript. All authors were actively involved in the editing of the manuscript.

## Conflict of Interest

The authors declare that the research was conducted in the absence of any commercial or financial relationships that could be construed as a potential conflict of interest.
